# Symbiotic intelligence in dental trauma diagnostics—an exploratory case study

**DOI:** 10.3389/froh.2025.1687841

**Published:** 2026-01-15

**Authors:** Rune Johan Krumsvik, Kristin Klock, Magnus Holmøy Bratteberg

**Affiliations:** 1 Department of Education, Faculty of Psychology, University of Bergen, Bergen, Norway; 2 Department of Clinical Dentistry, Faculty of Medicine, University of Bergen, Bergen, Norway

**Keywords:** case study, dental education, generative artificial intelligence, remote rural areas, symbiotic intelligence, traumatology

## Abstract

Dental trauma in children is common and requires prompt diagnosis, which can be challenging in remote or isolated settings with limited access to emergency dental care. This exploratory case study investigates whether OpenAI's o3 can support dental trauma diagnostics in primary incisors, building on prior pretesting of GPT-4 on summative dental education exams (2023) and multimodal dental trauma analyses (2024), and focusing on o3's multimodal capability and reliability in 2025 with expert assessment (“human in the loop”) prior to a supervisor seminar with students and supervisors (*N* = 84). Preliminary findings indicate that GPT-4 performed well on sample exams (2023), and that 7/10 multimodal analyses of dental injuries were accurate (2024); in the 2025 case, o3 correctly identified pulp necrosis in tooth 51 and uncomplicated enamel/dentin fractures in teeth 51 and 61, consistent with IADT guidance. Human expert involvement contributed essential validation, particularly for treatment decisions and ethical considerations. Overall, the study illustrates how symbiotic intelligence—purposeful collaboration between human and AI—may enhance learning outcomes in scenario-based simulations in remote areas, while requiring active human involvement and multiple validation communities.

## 1 Introduction

Traumatic injuries to the primary incisors affect roughly 30% of children under six-year-olds (1, 2), occurring at home, in kindergartens, and during leisure activities. Rapid diagnosis is crucial to prevent infection and permanent damage (3). At the same time, timely access to a dentist can be particularly challenging in sparsely populated areas, rural communities isolated by landslides, or during societal lockdowns (e.g., pandemics), where urgent dental care may be difficult or impossible to obtain. Such scenarios warrant greater emphasis in dental education, given the frequently noted learning gap between theoretical knowledge and clinical decision-making among dental students (4).

**Figure 1:**
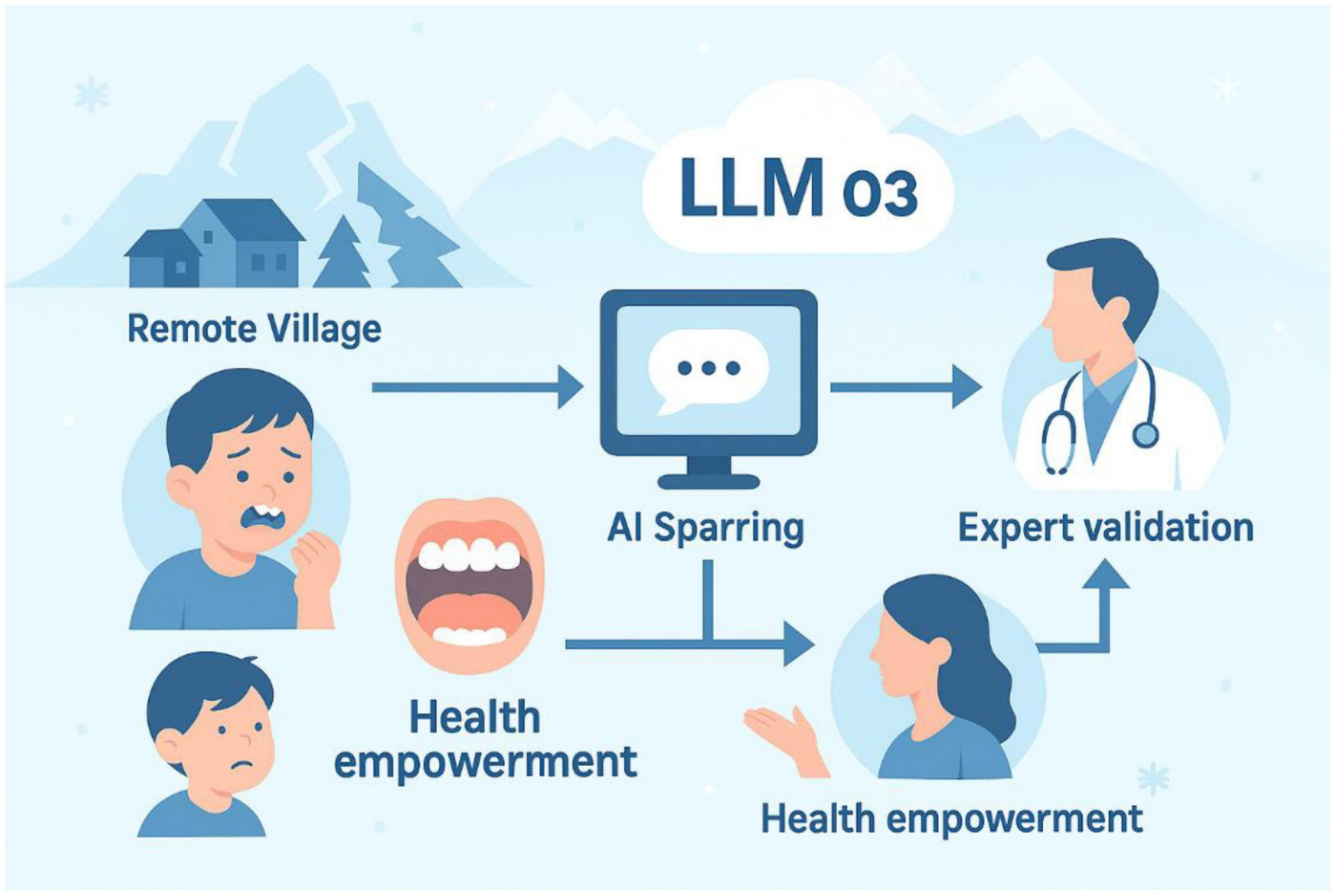
An illustration of the case study (original image created by the authors using OpenAI's o3 model).

Harte et al. (5) argue that the traditional undergraduate dental curriculum is at a turning point and must adapt to an era in which artificial intelligence plays an increasingly central role. They emphasize that dental students need not only to learn how to use AI tools, but also to develop a clear understanding of the limitations of such technologies. Moreover, the authors highlight that AI education should not replace established pedagogical approaches; rather, it should be integrated in a way that complements and strengthens the existing dental curriculum. Over the past two years, generative language models—exemplified by GPT-4, GPT-4o, and o3—have emerged as dynamic learning tools capable of providing immediate, case-based feedback in both dental education (4) and medical education (6). A growing body of knowledge syntheses (7), randomized controlled trials (8), and intelligence-test studies (9) indicates that state-of-the-art AI models can function as sparring partners and as part of a broader validation ecosystem for students and professionals alike (10). Nonetheless, their seemingly impressive predictive abilities raise questions about hallucinations, privacy, ethical responsibility, GDPR (General Data Protection Regulation), plagiarism, and assessment practices—suggesting that “human in the loop” vs. “AI in the loop” is not an either–or, but a both–and. Symbiotic intelligence (11, 12) is a tentative conceptual framework in which human clinical judgment and AI capability are iteratively combined to achieve better outcomes than either could alone. In dentistry, such collaboration may strengthen students’ learning processes and diagnostic skills—without compromising patient safety—through simulations and case work in dental education. It may also lay the groundwork for assessing whether AI can, in fact, enhance health empowerment among people in remote areas without immediate access to dentists or dental services (Figure 1).

Against this background, the aim of this simulated case is to examine whether o3 can function as a sparring partner in diagnosing an acute dental injury to the primary incisors, while also illuminating the role of the human-in-the-loop within a symbiotic-intelligence setting. The context is an annual supervisor seminar for dental students and supervisors (*N* = 84) held prior to a placement in The Public Dental Health Service, where similar themes have been addressed in earlier case studies (13–16). The research question is: *To what extent is o3 capable of performing a diagnostic assessment of an injury to the primary incisors?*

## 2 Method

### 2.1 Design

This pilot study is an exploratory, intrinsic case study (17, 18) conducted in spring 2025 at a Faculty of Medicine; Institute of Clinical Dentistry in Norway. The research-ethical aspects were assessed by the investigators and classified as a simulated case (no patient data) and educational development, with no requirement for formal ethics approval from SIKT (Norwegian Agency for Shared Services in Education and Research) or REK (Regional Committees for Medical and Health Research Ethics). Nevertheless, an ethical approval procedure was conducted to ensure that the simulated case was as authentic and ethically robust as possible (see Ethics Statement). In addition, relevant Norwegian public information resources were consulted (23, 24).

The pilot builds on: (i) pre-testing of GPT-4's capability and reliability on spot samples from summative examinations in dental education (2023); (ii) pre-testing of GPT-4's capability for multimodal analysis of 10 dental injuries/trauma cases before, during, and after an annual supervisor seminar (2024). These represent the main categories of dental trauma typically encountered in clinical settings and are grouped into five main types: Enamel fractures, Enamel–dentin fractures (with or without pulp exposure), Luxation injuries (including concussion, subluxation, extrusion, lateral luxation, and intrusion), Avulsion and Root fractures. (iii) pre-testing of o3's multimodal analysis of a dental injury/trauma, together with an expert appraisal by an experienced dentist [“human in the loop,” (19)] of the o3 analysis prior to the 2025 annual supervisor seminar.

### 2.2 Context, participants and material

**Figure 2:**
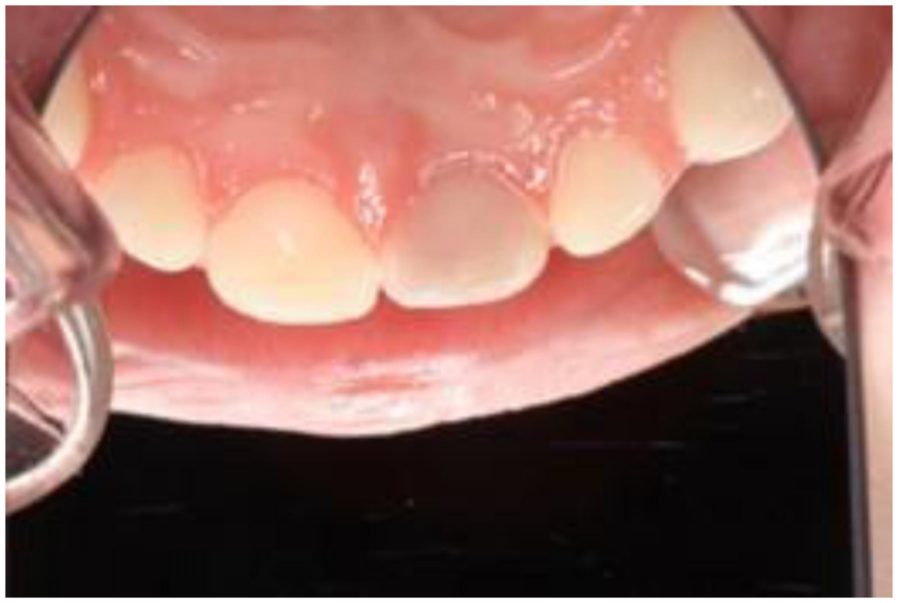
Intraoral photograph of a child's maxilla after dental trauma (photo: Anne Rønneberg).

A professor of pedagogy with advanced expertise in artificial intelligence, a senior professor of dentistry, an associate professor of dentistry with AI competence, and the o3 language model were the central actors in this pilot study. The study took place in the context of an annual supervisor seminar held for the twenty-fifth time, bringing together 44 fourth-year dental students and 40 experienced supervisiors from The Public Dental Health Service. Although students were not part of the sample in this case study, the seminar engaged both dental students and supervisors with a simulated scenario designed to prompt *reflection in action and reflection on action* (20). Through this process, participants were encouraged to draw on their own clinical experiences to identify parallels with the case, thereby engaging in *naturalistic generalization* (17). This approach is intended to foster the development of professional judgment by helping students carry insights from the case into their continued dental training, clinical placement, and eventual real-world practices. The case material consisted of a high-resolution intraoral photograph of a child's maxilla following dental trauma (Figure 2).

### 2.3 Chain-of-thought prompt—dental trauma

OpenAI's o3 (April 2025 release) was accessed via the ChatGPT interface (10th May), and the following chain-of-thought prompt was used to perform a multimodal analysis and assessment of the image:

You are an experienced dentist with specialist expertise in traumatic dental injuries in children. You receive a high-resolution intraoral photograph of a child's maxilla in which one primary central incisor appears grayish-discolored. Complete the task below by thinking out loud—write all intermediate steps, observations, and professional judgments before you give a conclusion.

Task:

Describe precisely what you see (color changes, anatomy, surrounding tissues, and any fractures).
List possible causes (differential diagnoses) of the observed discoloration.
Evaluate each cause against the clinical findings and likelihood—refer to relevant guidelines (3) and pathophysiology.
Formulate a tentative diagnosis and indicate your confidence (0%–100%).
Outline necessary additional examinations (clinical tests, radiographs, history) and explain why they are important.
Propose treatment options, including pros and cons, with particular emphasis on the patient's age and the risk to overall oral health.

First write your detailed thoughts (chain of thought) in full text. Then mark a clear section “Final conclusion” with a concise summary (max 3 sentences) that can be shared directly with a clinician or patient.

### 2.4 Procedure

The pilot study was conducted in seven phases:

Pre-testing (spot samples) of GPT-4 on summative dental examinations. From March–June 2023, GPT-4's capability and reliability were pre-tested using spot samples from summative examinations in dentistry. The results showed correct answers on 91% of the selected exam items.
Pre-testing of GPT-4 on clinical photographs of pediatric dental trauma. In February 2024, we pre-tested GPT-4's capability and reliability on 10 photographs of dental injuries in children. GPT-4 produced a correct diagnosis for 7 of 10 clinical photographs/cases.
o3's analysis of the dental trauma before the supervisor seminar. The researchers uploaded the image to o3 and conducted a dialogue in Norwegian requesting a clinical assessment and treatment plan. o3 received the prompt described above prior to the seminar and returned approximately a half-page analysis.
Expert review of o3's analysis by an experienced dentist. A senior clinician evaluated o3's write-up.
Researcher validation of the symbiotic-intelligence aspect. The research team decided how to present the case during the seminar to illustrate human–AI collaboration.
Supervisor seminar as a collective validation community. The case and tentative findings were presented at the seminar as a form of informal, naturalistic generalisation, where both students and supervisors validated the case *in situ*.
Comprehensive analysis of the pilot. Three researchers analysed and assessed the pilot in light of the research question and drafted a scholarly brief based on this work (the present Brief Research Report).

Accordingly, the study draws on a tentative literature synthesis, preliminary data from the 2023–2025 pre-testing, the o3 model's responses, and the expert dentist's appraisal. These materials were analysed as an exploratory intrinsic case study (17, 18) with a focus on (a) capability and reliability, (b) diagnostic accuracy, (c) student learning, (d) symbiotic intelligence, and (e) health empowerment.

## 3 Results

### 3.1 Diagnostic precision

Based on the chain-of-thought prompting described above (21), o3 made the following clinical observations from the image in Figure 2 (Table 1).

**Table 1:** O3s clinical observations from the image in Figure 2 (FDI tooth numbering).

Structure	Finding	Interpretation
51 (maxillary right primary central incisor)	Pronounced grey discoloration of the entire crown	Suggests likely pulp necrosis/internal bleeding after previous trauma
61 (maxillary left primary central incisor)	Normal colour, slight concave wear incisally	No obvious discoloration → likely vital; pulpal status intact
52/62 (laterals)	Light, uniform colour; no fracture	Vital; no signs of trauma
Gingiva/marginal tissues	No swelling or fistulae	No acute infection at present
Incisal edges 51/61	Small V-shaped irregularity in the middle of both	May be uncomplicated enamel/dentin fracture or physiological attrition

FDI, Fédération Dentaire Internationale tooth numbering system.

**Table 2:** O3 recommendations for further investigations and treatment (FDI tooth numbering).

Intervention	Purpose
Pulp sensibility tests (cold/electric)	Confirm vitality of 61 and adjacent teeth
Periapical radiograph of 51	Evaluate periapical changes, root resorption, and confirm pulp necrosis
Clinical follow-up	Monitor for discolored gingiva, sinus tract (fistula), or mobility
Extraction or pulpotomy (51)	Standard management of a necrotic primary incisor to prevent infection that could harm the permanent successor
Polishing/minor recontouring or composite for small enamel chips	Improve smoothness and aesthetics; reduce plaque retention

#### 3.1.1 Tentative diagnosis

Tooth 51—pulpal necrosis with post-traumatic internal discoloration. The grey colour change is classic for necrotic pulp in primary teeth after trauma. No signs of pulp exposure or deep fracture are visible in the image.
Possible uncomplicated crown injury (enamel ± dentin) on the incisal edges of 51 and 61. Small chips without visible pulp exposure. In the IADT classification, this corresponds to an uncomplicated crown defect (Ellis class I–II).

#### 3.1.2 Recommended further investigations and treatment

NB: Always consider the child's age, the time to natural exfoliation, and any symptoms when choosing between extraction and endodontic treatment of primary teeth.

In summary: The image (Figure 2) most strongly suggests that tooth 51 has lost vitality after a previous trauma and now shows characteristic grey discoloration, while the remaining teeth appear vital with only small, uncomplicated enamel defects. Further radiographic and clinical testing is required before a final diagnosis and treatment plan can be established (21).

o3 correctly proposed the primary diagnosis of pulp necrosis in 51, consistently identified internal discoloration as a sign of necrosis, and described the injury classification as an “uncomplicated crown defect” in accordance with IADT (2020). The experienced dentist confirmed that the AI's proposed diagnoses and reference to the Ellis classification were professionally valid. Post-lecture discussions with students and supervisors also indicated that they regarded the diagnosis as professionally sound.

### 3.2 Treatment proposals

o3 provided two alternative lines of treatment—extraction or pulpotomy—and emphasized the need for radiographs and sensibility testing. The experienced dentist pointed out that the AI did not mention specific contraindications (e.g., age, etc.) and that the time aspect of resorption should be discussed.

### 3.3 Learning experience

Students and supervisors gave generally positive feedback on the lecture of which this pilot study was an integrated part. The three researchers, the students, and the supervisors highlighted six main benefits: AI as a new, capable sparring partner; immediate feedback; breadth in differential diagnoses; a trigger for discussion; linguistic clarity; and relevance for health empowerment. Identified risks were information overload and potential blind trust in AI responses.

### 3.4 Human-in-the-loop dynamics

The validation communities identified some ambiguities in o3, particularly related to clarifying the differences between how one treats and follows up trauma in primary vs. permanent teeth. This matters for diagnostics, treatment choices, and for how students learn to assess different situations depending on the patient's age. It also provides an interesting angle on how AI tools can be better adapted—for example by accounting for age and dentition—and why “human in the loop” remains important in such cases. This aligns well with the goal of strengthening students’ clinical reasoning.

This case study shows that o3 can serve as support to improve diagnostic accuracy among dental students in acute trauma cases. The finding supports earlier reports that large language models can provide “personalized scaffolding” and formative assessment in health professions education (4, 6, 25). At the same time, the tentative findings reflect aspects of the theoretical framing of symbiotic intelligence (6, 11, 16), by demonstrating that suboptimal-to-optimal learning arises when human expertise (intellectual capacity, analytical ability, judgment, empathic assessment, practical constraints) is combined with the AI's “third eye,” knowledge generation, and depth reasoning around such cases.

## 4 Discussion

### 4.1 The importance of “human in the loop”

The absence of a competent and experienced dentist would likely have led to uncritical acceptance of AI claims that could entail some risk for student learning, even though this case does not involve an authentic patient case or patient treatment *per se*. This reflects the classic principle from Licklider (11) that humans should define goals and validate results while machines handle data-intensive routine tasks. Nevertheless, o3 takes this a step further and shows very strong capability and reliability in such simulated student learning situations. At the same time, it is important to remember that in a clinical setting, the subject-matter expert and supervisor constitute a necessary safety net that filters AI-generated knowledge through professional, ethical, legal, and contextual lenses. The case therefore indicates that symbiotic intelligence is an apt term for the theoretical framing of this pilot study (11, 12), which can be further developed by building a stronger evidence base going forward.

### 4.2 Implications for dental education

Based on the findings, we propose addressing a three-step design in the next part of the pilot study:

Independent reasoning—the dental student analyzes the case from scratch.
AI sparring—o3 is used to broaden differential diagnoses and propose treatment options; this is then discussed over time.
Expert validation—an experienced clinician discusses the AI output, highlights limitations, and provides final approval.

This process supports the FDI World Dental Federation (22) in developing AI-related digital competence among future dentists without delegating the responsibility for final clinical decisions.

### 4.3 Implications for health empowerment

**Figure 3:**
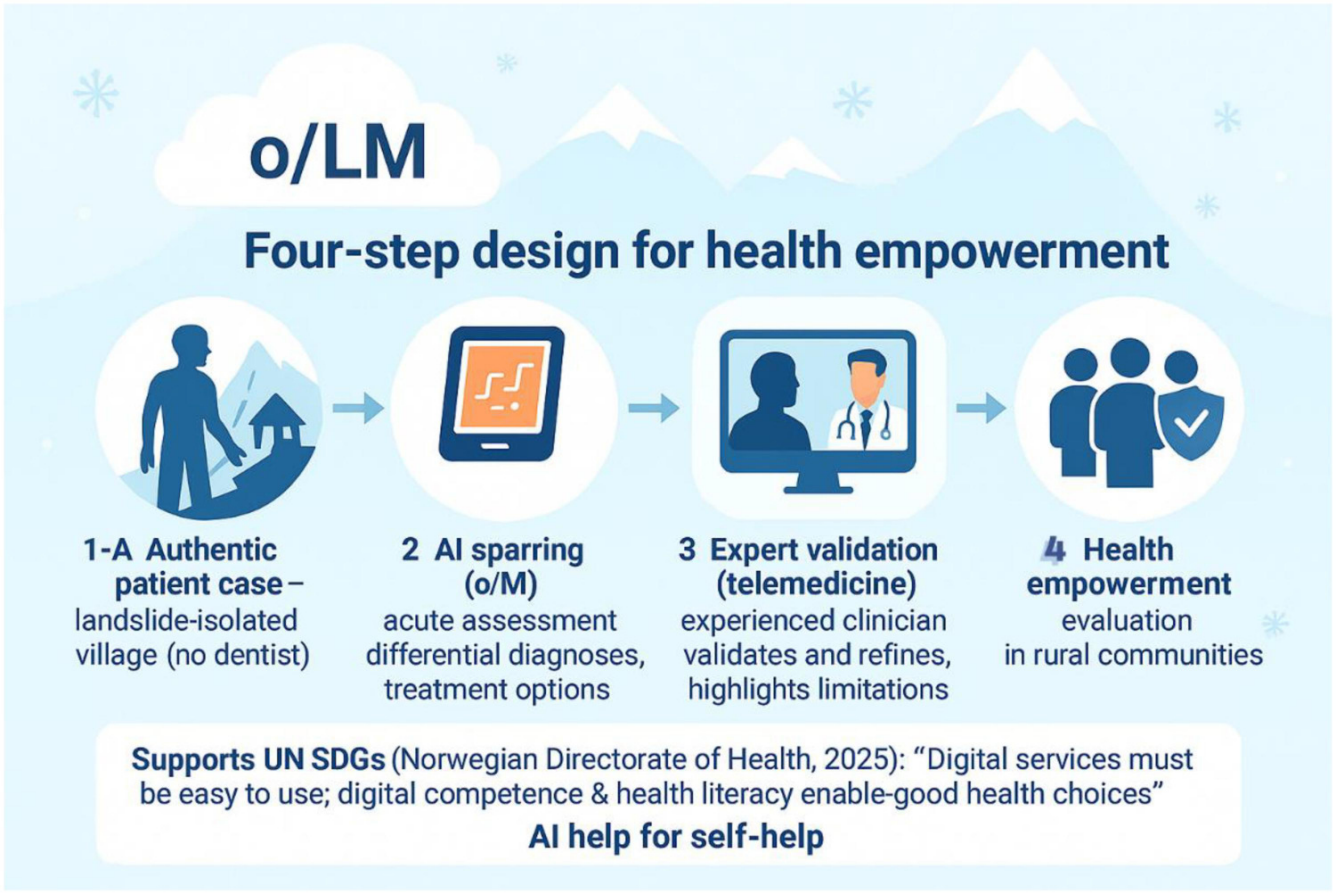
Four-step design for health empowerment in rural, landslide isolated villages (original image created by the authors using OpenAI's o3 model).

Based on the findings, we propose a four-step design in the next part of the pilot study (see Figure 3):

Authentic patient case—from a village cut off by a landslide in a remote rural area where a dentist or dental service is not available.
AI sparring—o3 is used to conduct an acute assessment, propose differential diagnoses and treatment options; this is then discussed locally over time through an interactive dialogue process with o3.
Expert validation—via video link and/or telemedicine, the dental injury is assessed by an experienced clinician who discusses o3's assessment, analyzes the dental injury via video/telemedical equipment, and highlights limitations and further treatment suggestions.
Health empowerment—an evaluation is conducted to determine whether this hinders or promotes health empowerment among people situated in such landslide-isolated, sparsely populated rural communities. According to the Norwegian Directorate of Health (2025), this development aligns closely with the UN Sustainable Development Goals. These goals emphasize that digital health services must be designed to be user-friendly, and that individuals need solid digital skills and sufficient health literacy to make informed decisions about their own health. In this way, the process contributes directly to empowering citizens and supporting sustainable health outcomes. This is particularly important in remote rural areas where “AI help for self-help” may strengthen health empowerment, though the evidence base is currently limited.

### 4.4 Contextual relevance

**Figure 4:**
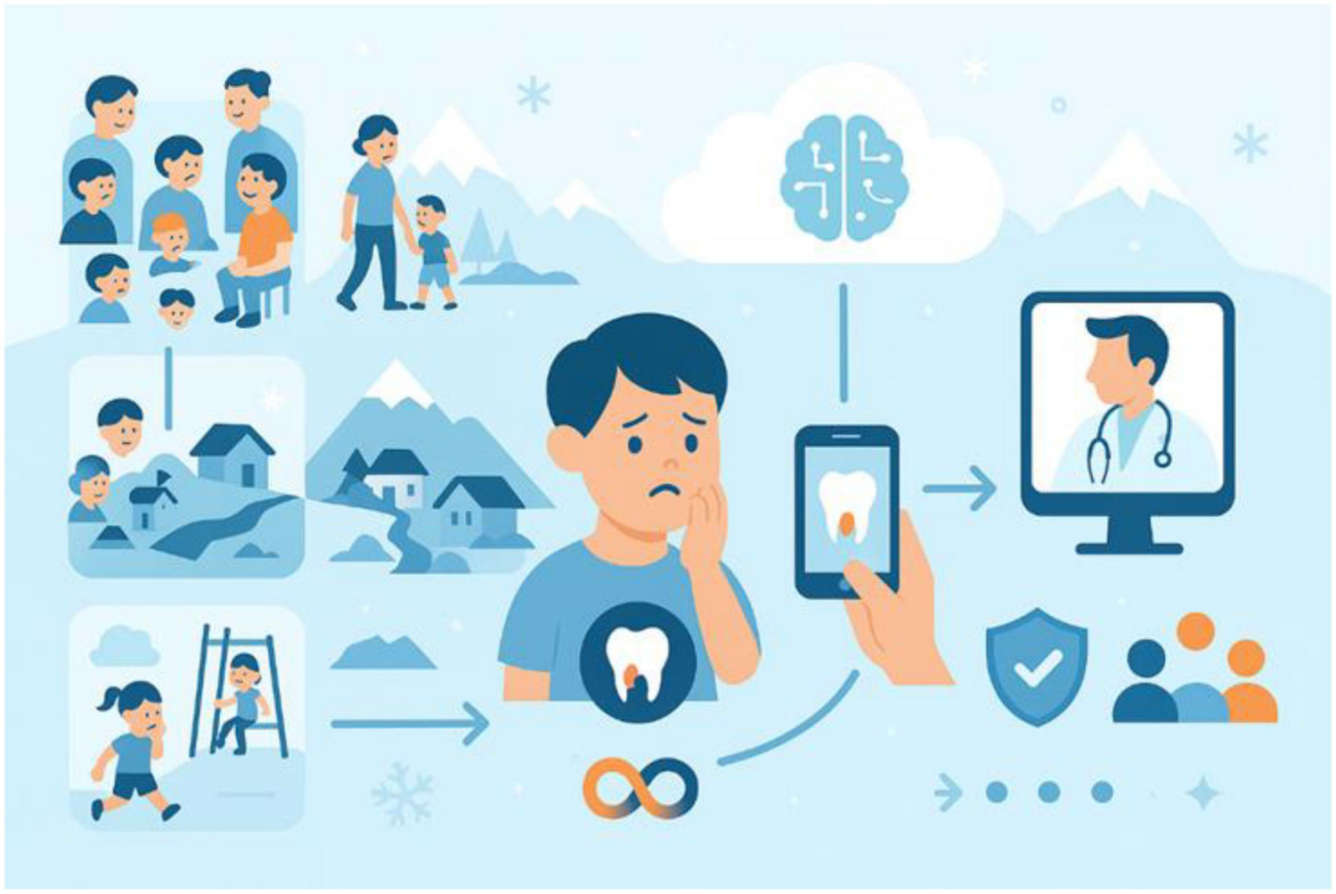
AI in scenarios where dental injuries occur in sparsely populated contexts, and how AI and telemedicine can strengthen health empowerment (original image created by the authors using OpenAI's o3 model).

Such acute trauma in primary incisors is widespread and frequent among children and adolescents and, in principle, requires rapid and correct diagnostics by dentists and the dental service. This is challenging for people living in remote rural areas, landslide-isolated communities, or during societal lockdowns (e.g., due to a pandemic), where access to emergency dental care is difficult or impossible (see Figure 4). This pilot study shows the importance of exploring the capability of AI in scenarios where dental injuries occur in sparsely populated contexts, and how AI (together with, for example, telemedicine, video consultations, etc.) can mitigate such situations and strengthen health empowerment. It may also help dental education—given the increasing requirement that dental students acquire AI competence and clinical reasoning early in their training—by giving students opportunities to engage with these types of cases in addition to their existing learning and practice arenas.

### 4.5 Limitations

The pilot study includes only one case of a clinical situation and two dentists, which imposes several limitations and restricts generalizability. Our interaction with GPT-4o and o3 took place in Norwegian; language-specific biases may have influenced the responses. Image quality is critical for GPT-4's and o3's capability and may be a source of error leading to inaccurate diagnoses in such cases. Furthermore, the two dentists were also researchers, which may introduce observer bias. Systematic data from students and supervisors were not collected during the supervision seminar regarding their validation of the case; thus, this rests on field dialogue and *naturalistic generalization* (17). This is also a limitation of the pilot study.

The rapid pace of system-level improvements in large language models such as GPT must be acknowledged as a limitation in this study. Model updates can occur within weeks or months, often leading to fewer errors, improved factual accuracy, multimodal capabilities and enhanced reasoning capabilities. In this study, GPT-4 was used during 2023–2024, while OpenAI's o3 model was applied in 2025. Consequently, some findings—particularly those concerning error rates, factual accuracy, and reasoning performance—should be interpreted as time-bound and may represent an upper limit relative to newer model iterations. This temporal variability also affects reproducibility and comparability across studies. To mitigate these challenges, we have (i) documented the exact model versions and access time, (ii) version-stamped the prompts and cases used, and (iii) planned follow-up replication studies with newer models to assess robustness and potential drift over time.

### 4.6 Future research

Future studies will build on the pretesting and be conducted in larger student groups and populations, with different cohorts, varied case types, and quantification of learning outcomes using validated mixed-methods research (MMR) data. There is also a need to develop an evidence base for guidelines that clearly define the division of responsibilities among students, AI tools, supervisors, and the public's health empowerment.

## 5 Conclusion

The research question for this pilot study was: *To what extent is o3 capable of diagnosing a dental injury in primary incisors?* In this case within dental education, o3 proved to be a capable and reliable sparring partner for diagnosing traumatic primary incisor injuries. A main conclusion is therefore that o3, under the conditions on which this case study is based, is capable of making an initial diagnosis of a dental injury in primary incisors (provided high-quality images). Considering the practical implications, however, such AI use for acute dental injuries in remote rural areas should be framed as a balance between AI-generated health empowerment and a human-in-the-loop model—which together safeguard patient safety better than either humans or AI alone. For dental education, this form of symbiotic intelligence may reduce the learning gap between theoretical knowledge and clinical decision-making among dental students (4) by integrating AI-supported clinical cases earlier in the curriculum (as summarized in Table 2). In this way, dental students also acquire digital competence during training that will strengthen their entry as new dentists into an increasingly AI-shaped society and professional context after graduation.

## Data Availability

The raw data supporting the conclusions of this article will be made available by the authors, without undue reservation.
